# Dynamic prediction of life-threatening events for patients in intensive care unit

**DOI:** 10.1186/s12911-022-02026-x

**Published:** 2022-10-22

**Authors:** Jiang Hu, Xiao-hui Kang, Fang-fang Xu, Ke-zhi Huang, Bin Du, Li Weng

**Affiliations:** 1 Medical Intensive Care Unit, Peking Union Medical College Hospital, Peking Union Medical College, Chinese Academy of Medical Sciences, 1 Shuai Fu Yuan, Beijing, 100730 China; 2 Hangzhou Maicim Medical Tech Co., Ltd, Hangzhou, Zhejiang China

**Keywords:** Early prediction, Deterioration, Life-threatening events, Mortality, Machine learning

## Abstract

**Background:**

Early prediction of patients’ deterioration is helpful in early intervention for patients at greater risk of deterioration in Intensive Care Unit (ICU). This study aims to apply machine learning approaches to heterogeneous clinical data for predicting life-threatening events of patients in ICU.

**Methods:**

We collected clinical data from a total of 3151 patients admitted to the Medical Intensive Care Unit of Peking Union Medical College Hospital in China from January 1st, 2014, to October 1st, 2019. After excluding the patients who were under 18 years old or stayed less than 24 h at the ICU, a total of 2170 patients were enrolled in this study. Multiple machine learning approaches were utilized to predict life-threatening events (i.e., death) in seven 24-h windows (day 1 to day 7) and their performance was compared.

**Results:**

Light Gradient Boosting Machine showed the best performance. We found that life-threatening events during the short-term windows can be better predicted than those in the medium-term windows. For example, death in 24 h can be predicted with an Area Under Curve of 0.905. Features like infusion pump related fluid input were highly related to life-threatening events. Furthermore, the prediction power of static features such as age and cardio-pulmonary function increased with the extended prediction window.

**Conclusion:**

This study demonstrates that the integration of machine learning approaches and large-scale high-quality clinical data in ICU could accurately predict life-threatening events for ICU patients for early intervention.

**Supplementary Information:**

The online version contains supplementary material available at 10.1186/s12911-022-02026-x.

## Background

Critically ill patients are admitted to Intensive Care Unit (ICU) for constant care and continuous organ support [1]. Frequent lab tests and medications are provided until patients are discharged. Patients who are admitted to ICU are very sick and therefore they tend to have a higher mortality rate than the average patients on the wards. With the development of artificial intelligence, many researchers have applied machine learning methods to study severe illnesses, such as sepsis [2–7], Acute kidney injury (AKI) [8–11], hypotension [12–14], etc. Early prediction of patients at high risk of deterioration within a short period of time would trigger immediate attention and intervention and thus would reduce the mortality rate at ICU, especially in low-and middle-income countries with limited health care resources [15–17].

Machine learning (ML) is a subfield of artificial intelligence that enables a computer to learn from data to make predictions, and the learning performance improves with more training data [18]. A variety of ML methods such as clustering analysis and pattern classification have been used in medical science. Clustering analysis is used to discover cluster structures within data in an unsupervised manner while pattern classification is based on labeled data (i.e., supervised learning). ML techniques can more effectively reduce errors and expenses compared with manual operation [19] and have better performance in prediction than traditional scoring methods, such as APACHE, SAPS, and SOFA [17]. Several studies were carried out to predict patient outcomes in ICU. For example, Gong et al. applied a logistic regression approach to predict patient outcomes based on the features extracted from the free-text notes in the Electronic Health Records (EHR) [20]; Johnson et al. predicted patient outcomes using a Bayesian Ensemble method [21]. However, these studies only included data within 72 h of ICU admission while excluding critically ill patients who stayed at ICU for over 3 days. Moreover, most machine learning models for ICU patients utilized a small number of features to predict the risk of mortality [22]. Given the high heterogeneity of patients at ICU, a limited number of features may not be able to capture some important signs of deterioration, leading to suboptimal performance. To better understand a prediction model and feature contributions, interpretable models were often utilized [23, 24]. For example, MAP, bicarbonate, and creatinine were found to be the top three important features contributing to predict acute gastrointestinal bleed [24].

So far, most of the published machine learning studies of ICU patients at ICU were based on an open database, Medical Information Mart for Intensive Care (MIMIC), which was established by MIT Institute of Computational Physiology, Beth Israel Deaconess Medical Center (BIDMC) and Philips Medical [25]. Few studies were carried out based on ICU data from China.

This study aims to develop a dynamic model for predicting life-threatening events, based on the data of the patients admitted to the Medical Intensive Care Unit of Peking Union Medical College Hospital (PUMCH-MICU).

## Methods

### Study population and data set

This study was based on the database of the Medical ICU in PUMCH. The dataset includes 3151 patients admitted to the PUMCH-MICU from January 1st, 2014, to October 1st, 2019. The patients who were less than 18 years old or stayed at ICU for less than 24 h were excluded. Five-fold cross-validation was employed to select samples for training and testing, i.e., the ratio of the sample size in a training set to a testing set is 4. The target events of our prediction included not only the outcome of mortality, as most similar studies did, but also predicted life-threatening events which happened during hospitalization. All cardiac arrest-related cardiopulmonary resuscitation was considered life-threatening event [26]. Therefore, in this study we defined life-threatening event as any signs of cardiac arrest, i.e., the operations of electric defibrillation or chest compression. Because palliative care was not an indication for ICU admission, all decedents received either electric defibrillation or chest compression. We used life-threatening event instead of cardiac arrest because chest compression usually started when the heart rate was declining. The data of vital signs and mechanical ventilation were automatically transferred to the database at intervals of 15 s and 1 min, respectively. The value of laboratory findings including blood gases were automatically collected. The inputs and outputs were manually recorded by registered nurses every hour. In this study, we collected data every hour on the hour and instant data at the life-threatening moments, which were manually confirmed by registered nurses. In addition, a statistic analysis was performed to detect the outliers. If a value was beyond the four standard deviations of the mean, the data would not be considered. In this study, the mean and standard deviation of the percentage of the feature value outliers in the whole cohort were 0.43% and 0.0047, respectively, suggesting high quality of the collected data.

### Feature selection

**Fig. 1:**
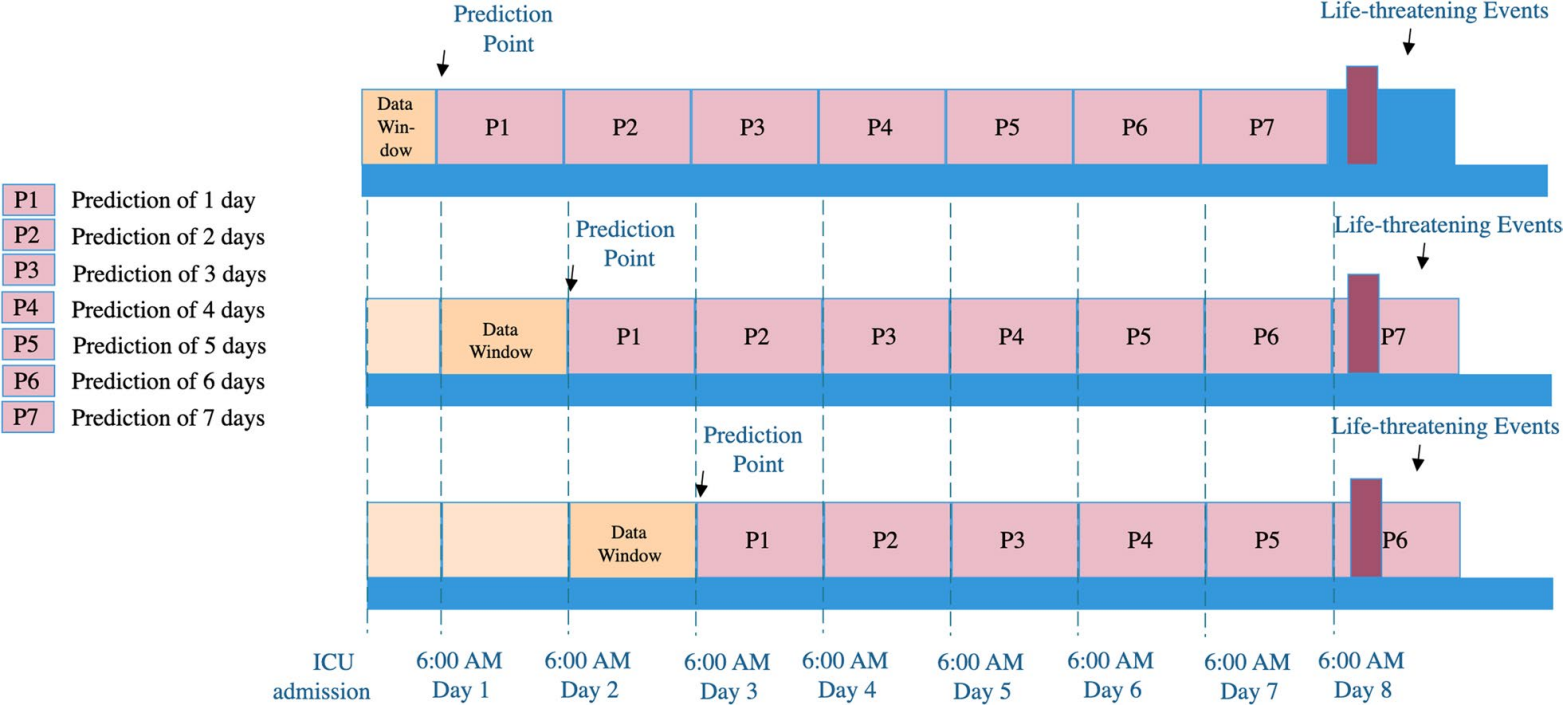
Schematic Diagram of Study design and model development. The prediction point was at 6:00 AM every day and the length of the prediction window was from 1 to 7 days. The 24-h observational data window was the period during which the data were collected for making predictions

Figure 1 illustrates our study design. A 24-h observational data window for each patient was used and the outcomes in the subsequent 7 days were determined. About 100 features (variables) were selected based on feature importance score computed by an embedded method Random Forest, and the features with importance score equal to zero were removed. The selected features were categorized into two groups, i.e., static features and dynamic features. Table 1 shows demographic features, vitals, lab testing results, hemodynamics, blood gases, inputs, outputs, and ventilator measurements. During each data window, vital, input and output features from each patient were collected hourly, and the tests for hemodynamic, lab, blood gas were usually measured once every several hours. Measurements of ventilators were only collected when available. For dynamic features, different methods were implemented to extract features. For hemodynamic, blood gas and vital features, and derivative features such as mean, max and min were computed. For each feature in the input and output categories, the sum of its values over 24 h was calculated.

**Table 1:** Static and dynamic features used in this study. These features are classified into eight categories

Category	Features (Variables)	Derivative Features	Static/dynamic
Demographic	Gender, cardiorespiratory function, CPR, Admission type, ARF, Shock, Coronary Artery Disease, Diabetes, Hypertension, COPD, NYHA class IV, Solid tumors(metastases), Lymphoma, Leukemia, ILD, dialysis, cirrhosis, hepatic failure, myeloma, Rheumatic immune disease, glucocorticoid, immunologic deficiency		Static
Vital	HR, SPO_2_, ADBP, ASBP, AMBP, Pulse, Resp, Temper, NDBP, NSBP, Glasgow coma scale (GCS), GCS Eye opening, GCS Verbal Response, GCS Motor Response, RASS, PAPm, CVP	Mean, Max, Min	Dynamic
Hemodynamic	SVRI, CI, BSA, SVR(Picco), SI(Picco), LVSW	Mean, max, Min	Dynamic
Blood Gas	pH, Ca, Cl, K, BE, Na, Lac, HCO3, tHb, AnionGap, Hct, sO*2*, pCO_2_, O_2_Hb, COHb, HHb, MetHb, Glu, pO*2*, PaO_2__FiO_2_	Mean, Max, Min	Dynamic
Lab	HGB, WBC, RBC, MCV, HCT, PLT, EOS_PERCENT, LY_PERCENT, NEUT_PERCENT, MONO_ABS, Creatinine, MONO_PERCENT, RDW_S, PCT, Ca, TCO_2_, Urea, PDW, Glu, TBil, ALT, DBil, INR, PT	Mean, Max, Min	Dynamic
Ventilator	FiO_2__Set, VTe, MVe, RR, Ppeak, PS, RR_Set	Mean	Dynamic
Input	Injection, Nasal Feeding, Intravenous Intake, Infusion pump related fluid input, Norepinephrine, Midazolam, Furosemide, Epinephrine	Sum	Dynamic
Output	Urine Volume, Stool count	Sum	Dynamic

*CPR* cardiac pulmonary resuscitation, *ARF* acute renal failure, *COPD* chronic obstructive pulmonary disease, *ILD* interstitial lung disease, *HR* heart rate, *SPO*_*2*_ pulse oximeter oxygen saturation, *ADBP* arterial diastolic blood pressure, *ASBP* arterial systolic blood pressure, *AMBP* arterial mean blood pressure, *NDBP* noninvasive diastolic blood pressure, *NSBP* noninvasive systolic blood pressure, *NMBP* noninvasive mean blood pressure, *GCS* Glasgow Coma Scale, *RASS* Richmond Agitation–Sedation Scale, *PAP* pulmonary artery pressure, *CVP* central venous pressure, *SVRI* systemic vascular resistance index, *CI *cardiac index, *PICCO* pulse indicator continuous cardiac output, *P_SV* PICCO_stroke volume, *BSA* body surface area, *P_SVR* PICCO systemic vascular resistance, *P_SI* PICCO stroke index, *LVSW* left ventricular stroke wok, *PCO*_*2*_ pressure of carbon dioxide, *Ca* calcium, *Cl* chloride, *K* potassium, *BE* base excess, *Na* sodium, *Lac* lactate, *tHb* total hemoglobin, *Hct* hematocrit, *sO*_*2*_ Oxygen saturation, *pCO*_*2*_ = pressure of carbon dioxide, *O*_*2*_*Hb *oxyhemoglobin, *COHb* carboxhemoglobin, *HHb* deoxyhemoglobin, *MetHb* methemoglobin, *Glu* glucose, *pO*_*2*_ pressure of oxygen, *PaO*_*2*_ arterial oxygen pressure, *FiO*_*2*_ fractional inspired oxygen, *HGB* hemoglobin, *WBC* white blood cell, *MCV* mean corpuscular volume, *HCT* hematocrit, *PLT* platelet, *EOS_PERCENT* percentage of eosinophil, *LY_PERCENT* percentage of lymphocyte, *NEUT_PERCENT* percentage of neutrophil, *MONO_ABS* monocytes absolute, *RDW* red cell distribution width, *PCT* procalcitonin, *TCO*_*2*_ total carbon dioxide, *PDW* platelet distribution width, *TBil* total bilirubin, *ALT *alanine aminotransferase, *DBil* bilirubin, *INR* international normalized ratio, *PT* prothrombin time, *VTe* tidal volume expiration, *MVe* minute volume expiration, *RR *respiratory rate, *Ppeak* peak pressure, *PS* pressure support, *RR_set* the set of respiratory_rate

### Imputation of missing values

In this study, different imputation approaches were applied for different types of variables. The mean imputation was used for demographic variables, and a sample and hold method was used for imputing variables of vital, blood gas, hemodynamic and lab.

### Model development

The model construction was shown in Fig. 1. At each prediction time point at 6:00AM of each day, seven predictors were developed to predict the life-threatening events. Four machine learning methods including Logistic Regression (LR), Support Vector Machine (SVM), Random Forest (RF), and light Gradient Boosting Machine (lightGBM) were employed to perform the prediction. LR is a generalized linear regression model and has a good fit for linear relationship between predictor and outcome variables [18]. SVM is a well-known machine learning algorithm for linear or nonlinear classification by choosing different kernel functions [18]. RF is an ensemble algorithm that consists of many weak learners and outputs the final decision by majority voting strategy or mean strategy [27]. lightGBM is a novel machine-learning algorithm with excellent performance. As a combination of tree-based learning algorithms and a gradient boosting framework, lightGBM is capable of handling large-scale data [28–30] and achieves good performance with small data as well [31]. The boosting algorithm is based on a set of weak learners. The residual loss function is minimized through the sequential trees. In addition, at the training stage a repeated k-fold cross-validation was used. At the testing stage, the combined estimator predicted the probability of life-threatening events by a majority voting strategy.

### Statistical analysis

The characteristics of static features in the training and testing sets are shown in Table 2. The continuous variables are presented as mean ± SD. The performance of the ensemble method was estimated by the metrics of Area Under the Receiver Operating Curve (AUROC/AUC). The 95% confidence intervals (CI) of AUC were calculated by bootstraping. In addition, sensitivity, specificity, and positive predictive value (PPV) were calculated under the proper cutoff point determined by Youden’s index [32]:

$${\text{YI}} = {\text{sensitivity }} + {\text{ specificity }} - 1{ }$$

**Table 2:** Characteristics of the static features in the dataset

	Training		Testing	
	Patients without life-threatening events	Patients with life-threatening events	Patients without life-threatening events	Patients with life-threatening events
	n = 1496	n = 274	n = 341	n = 59
Age (year, mean ± SD)	55 ± 19	62 ± 17	57 ± 18	59 ± 19
APACHE II score (mean ± SD)	19 ± 7	25 ± 8	18 ± 6	23 ± 6
*Gender*				
Female	702 (47)	117 (43)	140 (41)	21 (36)
Male	794 (53)	157 (57)	201 (59)	38 (64)
CPR	36(4)	15(9)	10(3)	4(7)
*Admission Type*				
Emergency non-operation	677 (81)	127 (93)	226 (78)	46 (92)
Emergency Operation	91 (11)	5 (4)	41 (14)	4 (8)
Elective non-operation	40 (5)	3 (2)	9 (3)	0 (0)
Elective Operation	23 (3)	1 (1)	15 (5)	0 (0)
Elective transferred	120	31	38	9
*Complications*				
ARF	192 (13)	70 (26)	37 (11)	10 (17)
Shock	503 (37)	169 (64)	160 (48)	42 (72)
*Comorbidities*				
Coronary Artery Disease	198 (13)	54 (20)	46 (13)	14 (24)
Diabetes	224 (15)	59 (22)	63 (18)	9 (15)
Hypertension	438 (29)	98 (36)	128 (38)	17 (29)
COPD	66 (4)	15 (5)	6 (2)	1 (2)
NYHA class IV	61 (4)	18 (7)	15 (4)	2 (3)
Solid tumors	98 (7)	25 (9)	26 (8)	1 (2)
Lymphoma	45 (3)	10 (4)	9 (3)	5 (8)
Leukemia	27 (2)	11 (4)	1 (0)	1 (2)
ILD	41 (3)	16 (6)	5 (1)	1 (2)
Dialysis	45 (3)	10 (4)	7 (2)	1 (2)
Cirrhosis	19 (1)	4 (1)	7 (2)	0 (0)
hepatic failure	9 (1)	2 (1)	1 (0)	0 (0)
Myeloma	11 (1)	2 (1)	3 (1)	0 (0)
Rheumatic disease	272 (18)	54 (20)	56 (16)	11 (19)
glucocorticoid	364 (24)	79 (29)	87 (26)	27 (46)
Immunologic deficiency	5 (0)	1 (0)	0 (0)	0 (0)

*APACHE Acute Physiology and Chronic Health Evaluation*

A cutoff point is determined when YI reaches the maximum.

## Results

**Fig. 2:**
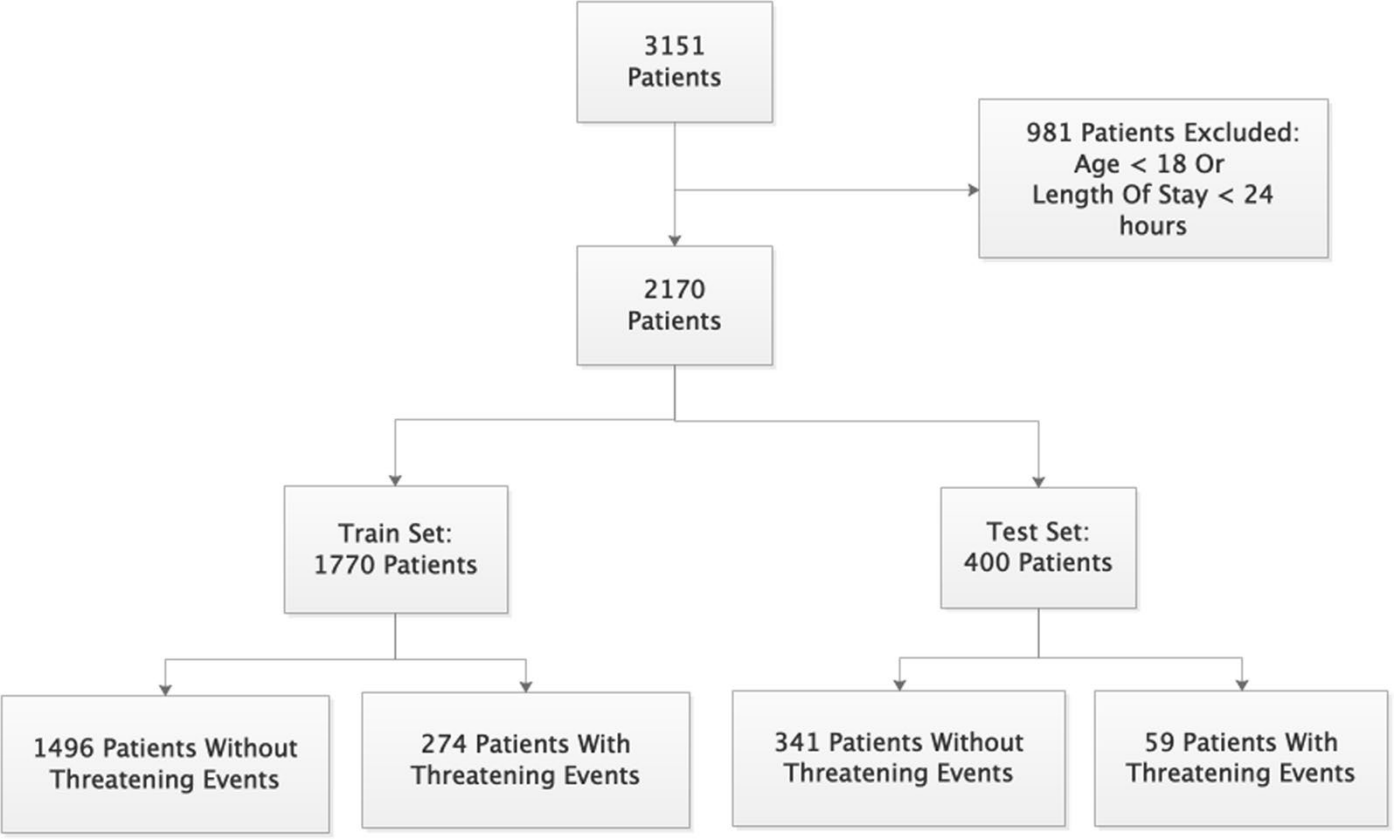
Flow chart of patient selection. From 3151 patients admitted to the PUMCH-MICU during a particular period of time, 2170 patients were selected for the subsequent analysis. The 2170 patients were then separated into a training set and a testing set for predicting life-threatening events

A total of 2170 patients admitted at PUMCH-MICU were included in the final analysis, as shown in Fig. 2. Most of the patients were admitted from the Department of Emergency Internal Medicine and 333 (15%) of the patients had life-threatening events during the study period.

### Characteristics of the static variables in the PUMCH-MICU dataset

Table 2 shows the characteristics of the static variables. The mean ages in the training and testing sets were 56(± 19) and 57(± 18), respectively.

### Determination of repeated k-fold cross-validation

**Fig. 3:**
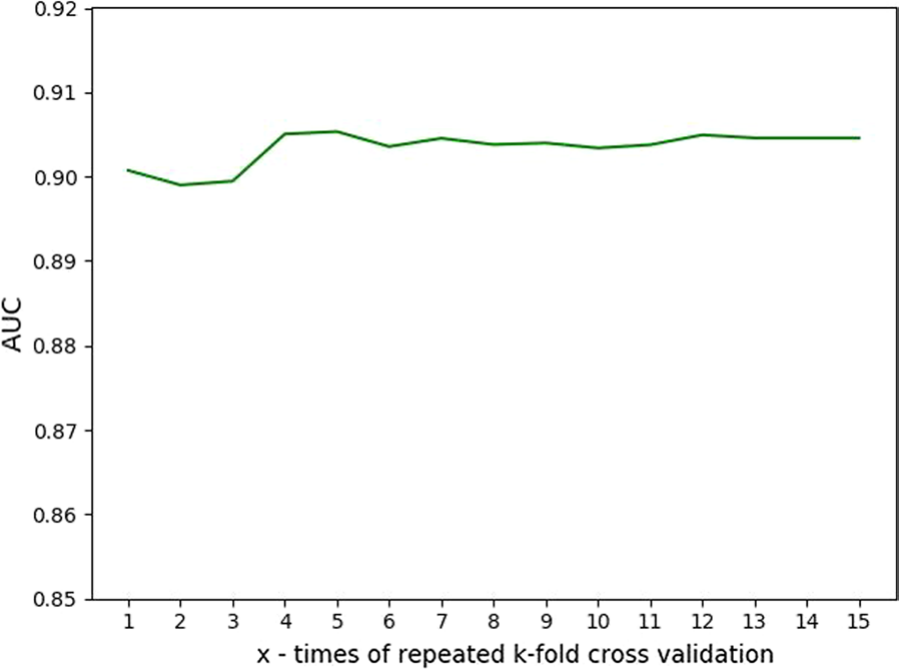
AUCs of GBM-D1 with various numbers of repetitions for k-fold cross-validation. The measured AUC of GBM-D1 varies as the number of k-fold cross-validation increases. The highest AUC of 90.5% was achieved with 4 repeatitions

As shown in Fig. 1, seven predictors were constructed to predict outcomes at different lengths of periods (i.e., day 1 to day 7) and were labeled as GBM-D1, GBM2, …, and GBM-D7. To find the best repeated times for k-fold cross-validation, we used GBM-D1 to test the performance with a range between 1 and 15 for repetitions. The results indicated that 4 repetitions for fivefold cross-validation achieved the best performance (Fig. 3).

### The performance comparison of four methods

**Fig. 4:**
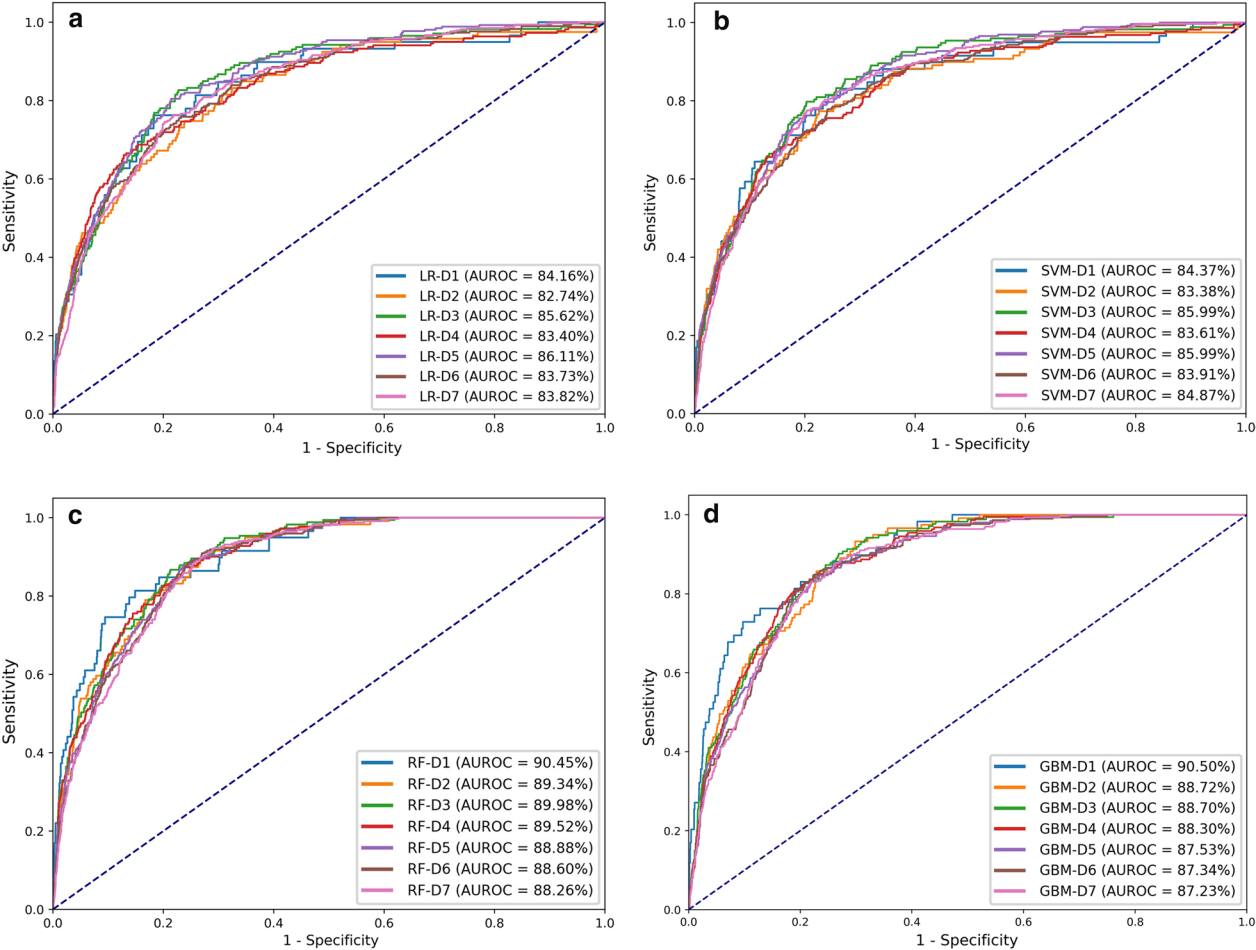
Performance comparison of four methods. These methods were applied to predict life-threatening events at seven different time periods, i.e., Day 1 to Day 7. The receiver operating curves of different models were plotted, and the AUCs were calculated. **a** Performance of Logistic Regression **b** Performance of Support Vector Machine **c** Performance of Random Forest **d** Performance of LightGBM

In this study, four different prediction methods were implemented, and their prediction performance was compared, as shown in Fig. 4. Both LR and SVM have an AUC of about 84% while RF and GBM had better performance (AUCs > 90%). Among the four methods, GBM had the best performance with an AUC of 90.5% on day 1. Therefore, we focused more on GBM in the subsequent analyses.

### Model development of life-threatening prediction

Finally, the performance of the seven predictors (GBM-D1, …, GBM-D7) on the testing data is shown in Table 3. As expected, GBM-D1 has the best performance with an AUC of 0.905 [95% CI 0.863, 0.930]). The AUC of GBM-D2 drops to 0.887 (95% CI 0.877, 0.922). The overall performance decreases gradually from 90.5 to 87.2% with the days increasing from 1 to 7. While GBM-D7 has the lowest AUC of 0.872 (95% CI 0.853, 0.887), it’s PPV of 0.231 is the highest (95% CI 0.195, 0.263).

**Table 3:** Performance comparison for the GBM based predictions. Sensitivity, Specificity and PPV are calculated under a predetermined threshold

	Threshold (Cutoff)(95% CI)	Sensitivity (95% CI)	Specificity (95% CI)	PPV (95% CI)	AUC (95% CI)
GBM-D1	0.374 (0.302, 0.437)	0.763 (0.703, 0.906)	0.872 (0.715, 0.915)	0.081(0.042, 0.131)	0.905 (0.863, 0.930)
GBM-D2	0.347(0.338, 0.464)	0.933 (0.785, 0.1)	0.702(0.656, 0.871)	0.087 (0.072, 0.168)	0.887 (0.877, 0.922)
GBM-D3	0.392(0.379, 0.488)	0.902 (0.782, 0.947)	0.731 (0.692, 0.855)	0.130 (0.110, 0.192)	0.887 (0.869, 0.905)
GBM-D4	0.450 (0.427, 0.466)	0.828(0.797, 0.894)	0.793(0.757, 0.821)	0.187 (0.159, 0.216)	0.883 (0.867, 0.899)
GBM-D5	0.455 (0.432, 0.5)	0.839 (0.761, 0.899)	0.776 (0.735, 0.843)	0.205 (0.18, 0.253)	0.875 (0.863, 0.895)
GBM-D6	0.451 (0.429, 0.503)	0.859 (0.77, 0.915)	0.764 (0.705, 0.83)	0.224 (0.182, 0.26)	0.873 (0.855, 0.888)
GBM-D7	0.441 (0.416, 0.474)	0.877 (0.801, 0.907)	0.738 (0.683, 0.784)	0.231 (0.195, 0.263)	0.872 (0.853, 0.887)

*CI* confidence intervals

### Feature importance

**Fig. 5:**
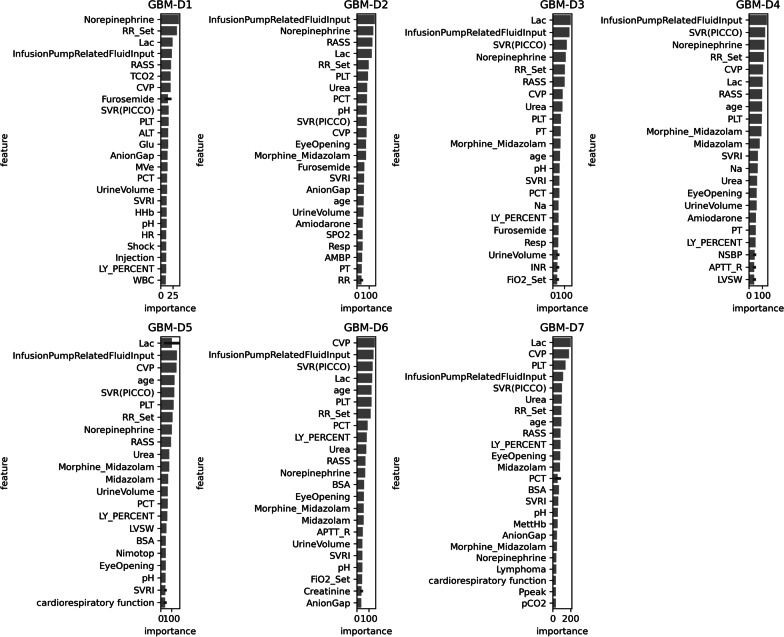
The ranking of features. The importance ranking is generated by lightGBM. The top 25 features are shown

As the lightGBM algorithm is based on tree-based weaker learners, it offers interpretable models. The top 25 most important features were shown in Fig. 5. Lac, Infusion pump related fluid input and CVP were the most important features in all the models. The ranking of norepinephrine decreases when the prediction window extends from day-1 to day-7. We also observed that the rankings of static features such as age, cardio-pulmonary function, and lymphoma increased as the prediction window extended.

### Sensitivity analysis

Considering that atrial fibrillation is not always life-threatening, defining electric defibrillation as a life-threatening event might not be accurate. As such, we performed a sensitivity analysis by excluding all the cases with electric defibrillation in the classification system. After excluding 29 cases of electric defibrillation, we found the main findings of our study remained robust as the AUC of GBM-D1 was 90.48% (the AUCs of these predictors see Additional file 1: Fig. S1).

As the different length of stay in ICU may cause the unbalanced number of samples for each patient, the predictors may be biased towards to those staying longer in ICU. To evaluate the impact of long ICU stays, a sensitivity analysis was performed on a dataset comprised of one positive sample and one negative sample randomly selected from each patient. The AUC of GBM-D1 was 90.58%, similar to the performance from the whole dataset (see Additional file 2: Fig. S2 for the plot of AUCs).

## Discussion

In this study, the algorithm, lightGBM, was employed to develop our models for predicting life-threatening events due to its superior performance in classification and ability of model interpretation. Single run of k-fold cross-validation might result in noisy estimate of performance, and repeated k-fold cross-validations could obtain reliable estimates [33].Thus, to reduce the randomness caused by cross-validation during training, the hyper-parameters were determined by a loop iteration.

Most studies of ICU patients’ prediction model were based on the data in the first 24–72 day of ICU admission to predict the 28-mortality [30] or in-hospital mortality [15, 17]. These prediction models provided few information on daily assessment and treatment. In this study, we constructed seven predictors for different periods from one day to seven days and included electric defibrillation and chest compression to provide more information in real world. Our predictors enabled dynamic surveillance of ICU patients and identified those at high risk. The metric, AUC, shows the performance decreased with the extended windows of prediction periods. Overall, we could predict life-threatening events for different periods with high accuracy. Given the high complexity of predicting patient outcomes at ICU, we evaluated 4 different prediction methods in the current study. In the actual application of the system, the best predictor will be utilized. Given different feature set and distribution of patient outcomes at different hospitals, we expect no single predictor be the best across all the hospitals and thus evaluation of different ML methods is critical for selecting the best one. GBM-D1 achieved the highest performance (AUC = 0.905 [95% CI 0.863, 0.930]) while GBM-D7 had the lowest performance (AUC = 0.872 [95% CI 0.853, 0.887]). When equal weights were assigned to the sensitivity and specificity, the optimal values can be determined by Youden’s index [34]. Instead of using the default value of 0.5 cutoffs, we applied Youden’s index to determine the cutoff values.

The ranking of features indicates the importance of each feature to prediction. In this study, the rankings of the top features were shown in Fig. 5. For GBM-D1, the top 5 features are norepinephrine, RR_set, Lac, Infusion pump related fluid input, and RASS. This ranking is not surprising as norepinephrine and infusion pump related fluid input are input events and RR_set is a parameter of the ventilator which is the intervention of treatment. A high value of lactate indicated the severity of a patient’s condition. Age was one of the most important features, and our results showed that age has greater weight in the predictors for a longer prediction window. The ranking of other static variables representing the fundamental condition of patients (e.g., cardio-pulmonary function and Lymphoma) increased with extension of prediction window. Therefore, our study revealed some fundamental conditions influencing the outcomes of ICU patients. However, model interpretation was crucial for clinicians to understand the rationale of prediction. The model we developed was used to predict short-term and medium-term outcomes of patients. For the bedside management of hemodynamic unstable patients with norepinephrine, our model might provide more predictive information (e.g., feature importance) about the probability of survival from day 1 to day 7 post norepinephrine initialization. The interpretable predictive model at each window is also crucial for clinicians to utilize the model at bedside. For example, SHapley Additive exPlanations (SHAP) [35] could be used to rank the import features for each prediction. Clinicians might pay more attention to the highest correlated features which are important for prediction when patients are at high risk. However, it remained a challenge in methodology to unlock black box in machine learning even in some common disease managment for intervention, i.e., Hypertension [36].

Our model includes a variety of features beyond the vital sign and represents the real-world condition of patients. To our knowledge, our study is one of the few studies investigating the possibility of predicting both short-term and medium-term life-threatening events for clinicians to decide on treatments and interventions.

There are several limitations in our study. First, the prediction was performed once for all the patients at one time point 6am every day before morning-up. This setup serves as an example to demonstrate the feasibility of the machine learning based prediction of patient outcomes. However, this model was not built for intervention once any risk factor was identified. Further investigation was urgently needed for any specifically detected high risk factors of an outcome of interest. In the actual clinical application, predictors will be easily retrained for predicting patient outcomes at every hour or at any timepoint (i.e., the real time prediction). In addition, the prediction will be done automatically for a patient whenever a new parameter from him/her comes in. The distribution of outcomes across the different time periods will affect the prediction performance but the impact can be minimized by properly selecting training samples at different classes. In the current study, the distribution of outcome is dynamic with average of approximate 4% of and standard variation of 0.054 for life-threntening events across all time points. Second, our study was based on a single center Medical ICU. Further validation using independent ICU patient cohorts are warranted to generalize the findings. However, the baseline characteristics of the patients in our study suggests that the model developed through this study is highly predictive of the critically-ill patients[16]. Furthermore. Goh et al. showed that the unstructured data can improve the model performance [7], and we will expand our feature set beyond the quantitative features in the future.

## Conclusion

In this study, dynamic predictors were developed to predict the risk of life-threatening events in ICU at seven different time points with high accuracy. Such predictions would help clinicians select treatments and interventions in both short term and medium term.

## Supplementary Information

**Additional file 1.** The AUCs of predictors after excluding the cases of electric defibrillation.

**Additional file 2.** The AUCs of predictors for randomly selecting one positive sample and one negative sample from each patient.

## Data Availability

The datasets generated and/or analyzed during the current study are not publicly available due to the confidentiality policy of Peking Union Medical College Hospital but are available from the corresponding author on reasonable request and with permission of Peking Union Medical College Hospital.

## Abbreviations

ALT: Alanine aminotransferase
ADBP: Arterial diastolic blood pressure
AMBP: Arterial mean blood pressure
ARF: Acute renal failure
ASBP: Arterial systolic blood pressure
AUC: Area under curve
BE: Base excess
BIDMC: Beth Israel Deaconess Medical Center
BSA: Body surface area
Ca: Calcium
CI: Cardiac index
Cl: Chloride
COHb: Carboxhemoglobin
COPD: Chronic obstructive pulmonary disease
CPR: Cardiac pulmonary resuscitation
CVP: Central venous pressure
DBil: Bilirubin
EOS_PERCENT: Eosinophil
FiO_2_: Fractional inspired oxygen
GBM: Gradient boosting machine
GCS: Glasgow Coma Scale
Glu: Glucose
Hct: Hematocrit
HCT: Hematocrit
HGB: Hemoglobin
HHb: Deoxyhemoglobin
HR: Heart rate
ICU: Intensive care unit
ILD: Interstitial lung disease
INR: International normalized ratio
K: Kalium
Lac: Lactate
LVSW: Left ventricular stroke work
LY_PERCENT: Lymphocyte
MCV: Mean corpuscular volume
MetHb: Methemoglobin
MIMIC: Medical Information Mart for Intensive Care
MONO_ABS: Monocytes absolute
MVe: Minute volume expiration
NA: Sodium
NDBP: Noninvasive diastolic blood pressure
NEUT_PERCENT: Neutrophil
NMBP: Noninvasive mean blood pressure
NSBP: Noninvasive systolic blood pressure
O_2_Hb: Oxyhemoglobin
PaO_2_: Arterial oxygen pressure
PAP: Pulmonary artery pressure
PCO_2_: Pressure of carbon dioxide
pCO_2_: Pressure of carbon dioxide
PCT: Procalcitonin
PDW: Platelet distribution width
PH: Potential of hydrogen
PLT: Platelet
pO_2_: Pressure of oxygen
Ppeak: Peak pressure
PS: Pressure support
PT: Prothrombin time
PUMCH-MICU: Medical Intensive Care Unit of Peking Union Medical College Hospital
RASS: Richmond Agitation–Sedation Scale
RDW: Red cell distribution width
ROC: Receiver operating curve
RR: Respiratory rate
RR_set: The set of respiratory rate
SI: Stroke index
sO_2_: Oxygen saturation
SPO_2_: Pulse oximeter oxygen saturation
SV: Stroke volume
SVR: Systemic vascular resistance
SVRI: Systemic vascular resistance index
TBil: Total bilirubin
TCO_2_: Total carbon dioxide
tHb: Total hemoglobin
VTe: Tidal volume expiration
WBC: White blood cell
PICCO: Pulse indicator continous cardiac output
