# Supplementary figures and images for: Dynamic prediction of life-threatening events for patients in intensive care unit
Source: BMC Med Inform Decis Mak. 2022 Oct 22;22:276. doi: 10.1186/s12911-022-02026-x (PMC9587604; doi:10.1186/s12911-022-02026-x)

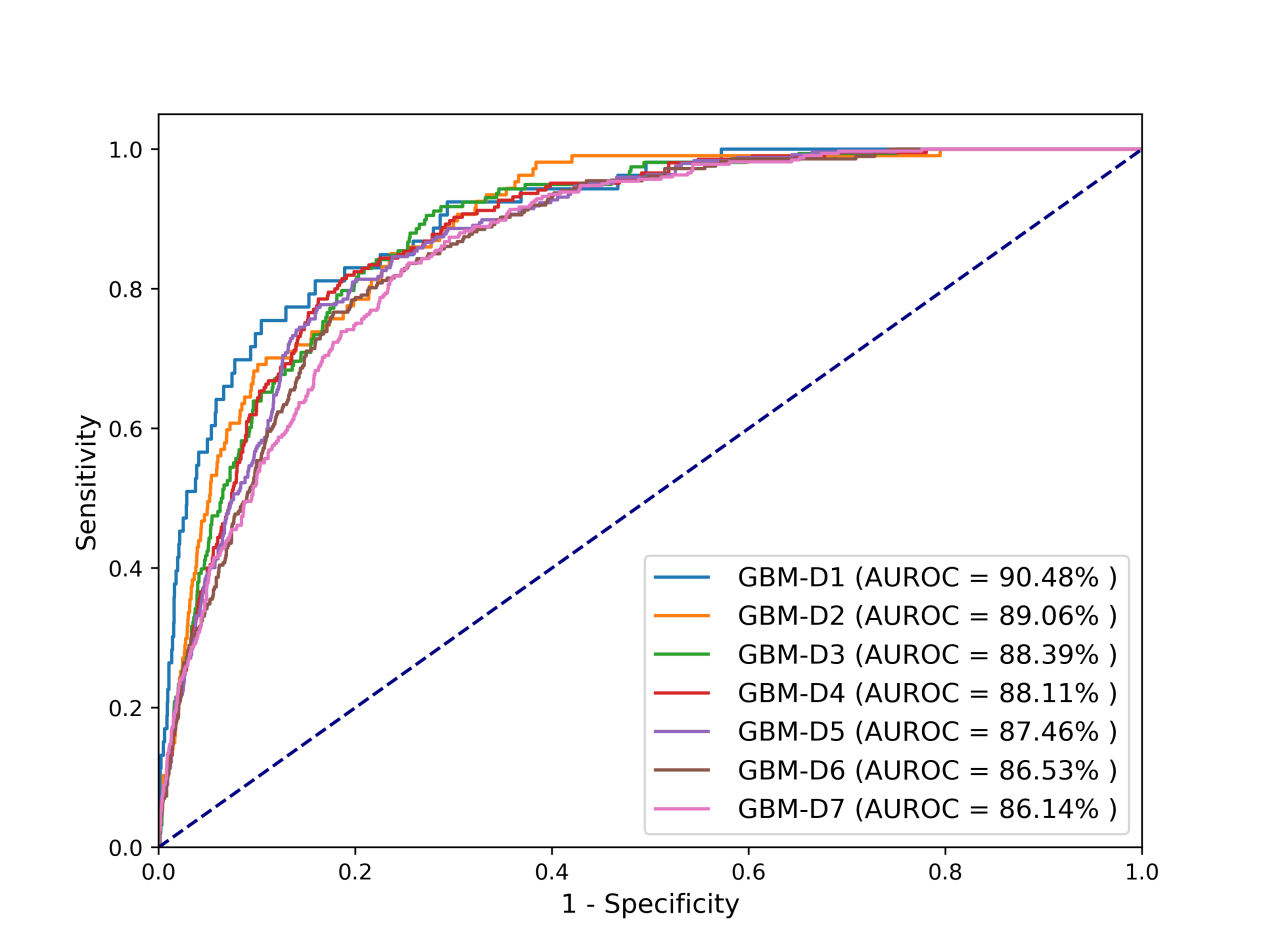


Figure S1: The AUCs of GBM-D1 to GBM-D7 after excluding 29 cases of electric defibrillation.

Supplement: Supplementary file 1 — Additional file 1. The AUCs of predictors after excluding the cases of electric defibrillation. [file 12911_2022_2026_MOESM1_ESM.docx]
