# Supplementary material for: Dynamic prediction of life-threatening events for patients in intensive care unit
Source: BMC Med Inform Decis Mak. 2022 Oct 22;22:276. doi: 10.1186/s12911-022-02026-x (PMC9587604; doi:10.1186/s12911-022-02026-x)
Supplement: Supplementary file 2 — Additional file 2. The AUCs of predictors for randomly selecting one positive sample and one negative sample from each patient. [file 12911_2022_2026_MOESM2_ESM.docx]

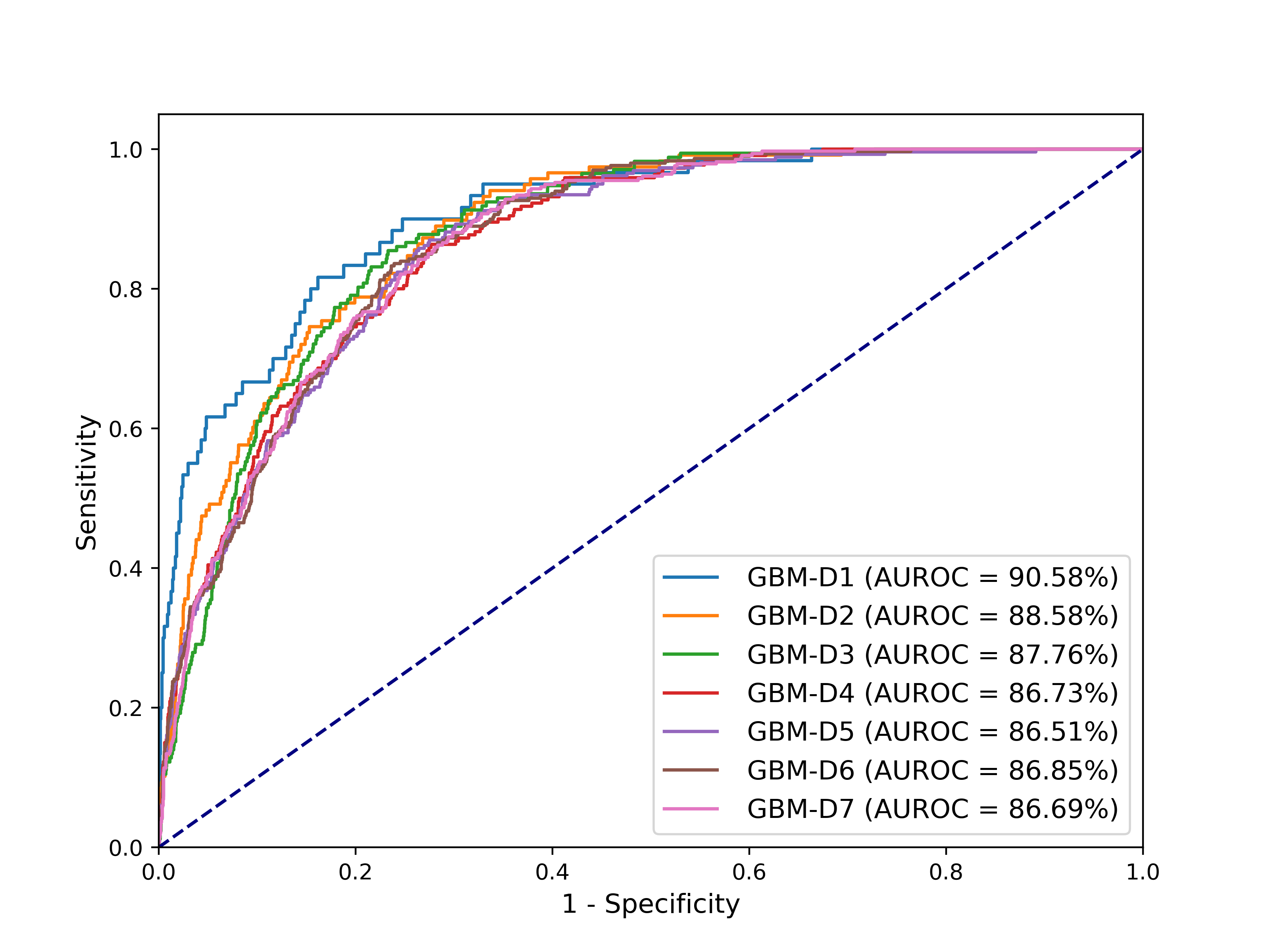


Figure S2: AUCs of GBM-D1 to GBM-D7 for the data comprised of one positive sample and one negative sample randomly selected from each patient.

。
